# Nonsurgical management of refractory pneumothorax: efficacy of blood patching

**DOI:** 10.1093/omcr/omab136

**Published:** 2022-01-24

**Authors:** Keigo Kobayashi, Akihiko Ogata, Toshiyuki Hirano, Takahiro Asami, Takashi Inoue

**Affiliations:** Internal Medicine, Sano Kosei General Hospital, Tochigi, Japan; Division of Medical Oncology, National Cancer Centre Singapore, Singapore, Singapore; Internal Medicine, Sano Kosei General Hospital, Tochigi, Japan; Internal Medicine, Sano Kosei General Hospital, Tochigi, Japan; Internal Medicine, Sano Kosei General Hospital, Tochigi, Japan; Internal Medicine, Sano Kosei General Hospital, Tochigi, Japan

## CLINICAL PICTURES

Pneumothorax rarely develops in patients with interstitial fibrosis, unless additional risk factors coexist, but when it does occur, it is often refractory to resolution with conservative management with a chest tube only [1]. The underlying mechanism of such pneumothoraxes is considered to be the result of the rupture of subpleural cystic spaces (bulla and bleb) related to diffuse interstitial fibrosis [2].

A 72-year-old man with combined pulmonary fibrosis and emphysema (CPFE) was hospitalized with his first presentation of severe closed pneumothorax. He had suffered from severe pulmonary hypertension (PH, mean pulmonary artery pressure 72.6 mmHg). We inserted a chest tube (a thoracic catheter, double-lumen, 20Fr) from the left fourth intercostal on the midclavicular line. We monitored the pleural drainage for 1 week; however, the air leak continued. We were unable to perform surgery on this patient because of his severe PH. His CPFE was also contraindications for other stimulant pleurodeses such as OK-432 [1]. Therefore, we tried to stop the air leak with autologous blood patch pleurodesis (ABPP) [3], which is an alternative to surgery when conservative management is ineffective for treating a persistent air leak. This is obtained by instillating 100 ml of autologous blood through the chest tube, followed by clamping 1 hour [4]. We repeated the chest X-ray every day to assess the state of pneumothorax.

**Figure 1:**
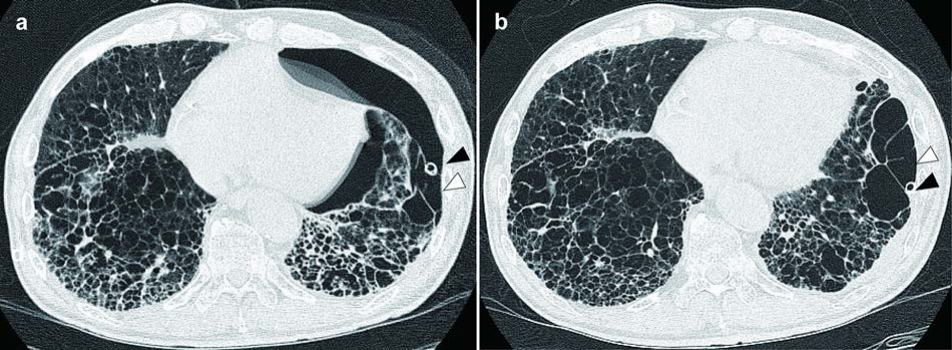
Chest computed tomography images: (**a**) after placing the second chest tube, the chest tube was placed more precisely over the air leak site; (**b**) after another attempt at ABPP, air leak had stopped and the left lung was fully inflated. Black arrow: chest tube tip, white arrow: bullae.

After ABPP was performed three times in total with an interval of several days, the air leak stopped and we removed the chest tube. However, 7 days after removing the tube, his pneumothorax recurred. Chest computed tomography indicated that the large bullae in his lower lung lobe were ruptured. We placed a chest tube from his eighth intercostal on the midaxillary line, aiming for more precise chest tube placement over the large bullae (Fig. 1a). We tried ABPP once again and finally succeeded in stopping the air leak (Fig. 1b). His pneumothorax had not recurred from >4 weeks after chest tube removal.

ABPP is a simple, safe and painless method for treating refractory pneumothorax; however, it may be important to adjust the chest tube placement to be precisely over the air leak site.
